# Resveratrol Contrasts LPA-Induced Ovarian Cancer Cell Migration and Platinum Resistance by Rescuing Hedgehog-Mediated Autophagy

**DOI:** 10.3390/cells10113213

**Published:** 2021-11-17

**Authors:** Alessandra Ferraresi, Andrea Esposito, Carlo Girone, Letizia Vallino, Amreen Salwa, Ian Ghezzi, Suyanee Thongchot, Chiara Vidoni, Danny N. Dhanasekaran, Ciro Isidoro

**Affiliations:** 1 Laboratory of Molecular Pathology, Department of Health Sciences, Università del Piemonte Orientale “A. Avogadro”, Via Solaroli 17, 28100 Novara, Italy; alessandra.ferraresi@med.uniupo.it (A.F.); andrea.esposito@uniupo.it (A.E.); carlo.girone@uniupo.it (C.G.); letizia.vallino@uniupo.it (L.V.); amreensalwa12@gmail.com (A.S.); 20020210@studenti.uniupo.it (I.G.); suyanee.thongchot@gmail.com (S.T.); chiara.vidoni@med.uniupo.it (C.V.); 2 Siriraj Center of Research Excellence for Cancer Immunotherapy (SiCORE-CIT), Research Department, Faculty of Medicine Siriraj Hospital, Mahidol University, Bangkok 10700, Thailand; 3 Stephenson Cancer Center, The University of Oklahoma Health Sciences Center, Oklahoma City, OK 73104, USA; Danny-Dhanasekaran@ouhsc.edu

**Keywords:** autophagy, tumor microenvironment, cell migration, epithelial to mesenchymal transition, BMI-1, 3D spheroids, transcriptomic, chemoresistance, overall survival, personalized cancer therapy

## Abstract

**Background**: Ovarian cancer progression and invasiveness are promoted by a range of soluble factors released by cancer cells and stromal cells within the tumor microenvironment. Our previous studies demonstrated that resveratrol (RV), a nutraceutical and caloric restriction mimetic with tumor-suppressive properties, counteracts cancer cell motility induced by stromal IL-6 by upregulating autophagy. Lysophosphatidic acid (LPA), a bioactive phospholipid that shows elevated levels in the tumor microenvironment and the ascites of ovarian cancers, stimulates the growth and tissue invasion of cancer cells. Whether LPA elicits these effects by inhibiting autophagy and through which pathway and whether RV can counteract the same remain obscure. **Aims**: To investigate the molecular pathways involved in LPA-induced ovarian cancer malignancy, particularly focusing on the role of autophagy, and the ability of RV to counteract LPA activity. **Results**: LPA stimulated while RV inhibited ovarian cancer cell migration. Transcriptomic and bioinformatic analyses showed an opposite regulation by LPA and RV of genes linked to epithelial-to-mesenchymal transition (EMT) and autophagy with involvement of the PI3K-AKT, JAK-STAT and Hedgehog (Hh) pathways. LPA upregulated the Hh and EMT members GLI1, BMI-1, SNAIL-1 and TWIST1 and inhibited autophagy, while RV did the opposite. Similar to the inhibitors of the Hh pathway, RV inhibited LPA-induced cancer cell migration and 3D growth of ovarian cancer cells. *BMI-1* silencing prevented LPA-induced EMT, restored autophagy and hampered cell migration, resembling the effects of RV. TCGA data analyses indicated that patients with low expression of Hh/EMT-related genes together with active autophagy flux tended to have a better prognosis and this correlates with a more effective response to platinum therapy. In *in vitro* 3D spheroids, LPA upregulated BMI-1, downregulated autophagy and inhibited platinum toxicity while RV and Hh inhibitors restored autophagy and favored BAX-mediated cell death in response to platinum. **Conclusions**: By inhibiting the Hh pathway and restoration of autophagy, RV counteracts LPA-induced malignancy, supporting its inclusion in the therapy of ovarian cancer for limiting metastasis and chemoresistance.

## 1. Introduction

Ovarian cancer represents the eighth-most diagnosed cancer among women worldwide and it accounts for the highest mortality rate among all the gynecological malignancies, with a 5-year survival rate less than 40% [1]. This has been attributed to its asymptomatic onset and an aggressive metastatic potential, which often results in the colonization of the peritoneal cavity and of the omentum [2]. In the majority of the cases, cytoreductive surgery and chemotherapy elicit an initial good response, which however is followed by tumor recurrence of chemoresistant clones that ultimately negatively impact on patients’ survival outcome [3].

Within the tumor microenvironment, the crosstalk between cancer cells and stromal cells, mediated by the exchange of metabolites and the secretion of growth factors and cytokines, impinges on the signaling pathways that control autophagy, cell proliferation and migration, thus creating the conditions for ovarian cancer progression [4,5].

Autophagy is a catabolic process devoted to the degradation of damaged or redundant cellular components within the lysosomes, and it plays a crucial role in macromolecular turnover and in maintaining cell homeostasis [6]. Deregulation of autophagy has been recognized as one of the main metabolic features of ovarian cancer cells subjected to changes in the tumor microenvironment in terms of nutrients, oxygen, growth factors, cytokines, non-coding RNAs and other signaling molecules [4,7,8,9].

In our previous studies we showed that resveratrol (RV), a nutraceutical and caloric restriction mimetic, is a formidable enhancer of autophagy [10] capable of counteracting the pro-invasive activity of IL-6 found in the tumor microenvironment [11,12]. 

Lysophosphatidic acid (LPA), a lipid growth factor highly secreted in the ovarian tumor microenvironment [13], exerts several pro-tumorigenic activities such as (i) reprogramming toward a glycolytic shift that associates with the pheno-conversion of ovarian normal fibroblasts into cancer-associated fibroblasts (CAFs) [14] and with the enhanced proliferation of ovarian cancer cells [15], (ii) upregulating the oncogenic/growth-promoting transcriptome [16,17], (iii) promoting EMT and invasiveness [18,19], (iv) stimulating the secretion of pro-inflammatory and angiogenic factors (such as TNF-α, IL-6, IL-8, VEGF, etc.) [20,21], and (v) promoting therapy resistance either by supporting an immunosuppressive phenotype or by protecting tumor cells from apoptosis [21]. Additionally, it has been reported that LPA inhibits autophagy in prostate cancer cells [22,23]. 

Here, we show that LPA promotes EMT and migration, 3D spheroid growth and platinum resistance in ovarian cancer cells and that RV could effectively counteract all these effects. Transcriptomic and bioinformatic analyses revealed the involvement of the Hedgehog (Hh) pathway in such effects. Mechanistically, LPA inhibited while RV, like Hh inhibitors and *BMI-1* silencing, rescued autophagy. From a clinical translational point of view, upregulation of the Hh pathway along with downregulation of autophagy associates with platinum resistance and poor prognosis in ovarian cancer patients. 

The present findings have clinical implications supporting the use of the autophagy enhancer RV as an adjuvant therapeutic for limiting metastasis and increasing chemoresponsiveness of ovarian cancer and thus improving patients’ clinical outcome.

## 2. Materials and Methods

### 2.1. Cell Culture

SKOV3 and OVCAR3 human ovarian cancer cells were cultured in RPMI 1640 medium (cod. R8758; Sigma Aldrich, St. Louis, MO, USA) supplemented with 10% heated-inactivated FBS (cod. ECS0180L; Euroclone, Milan, Italy), 1% glutamine (cod. G7513; Sigma Aldrich) and 1% penicillin/streptomycin (cod. P0781; Sigma Aldrich). 

OAW42 human ovarian cancer cells were cultured in MEM medium (cod. M2279; Sigma Aldrich) supplemented with 10% heated-inactivated FBS (cod. ECS0180L; Euroclone), 1% glutamine (cod. G7513; Sigma Aldrich), 1% non-essential amino acids (cod. M7145; Sigma Aldrich) and 1% penicillin/streptomycin (cod. P0781; Sigma Aldrich). 

SKOV3-GFP-LC3 cells were established in our laboratory and maintained in culture conditions as parental SKOV3 cells. The cells were transfected with the plasmid encoding for GFP-LC3 (as previously described in [24]) and clones stably expressing the transgenic chimeric DNA were selected upon two weeks culture in medium containing 1 mg/mL G418 disulfate salt solution (cod. G8168; Sigma Aldrich).

### 2.2. Reagents 

1-Oleoyl lysophosphatidic acid sodium salt (LPA, cod. 22556-62-3; Cayman Chemical, Ann Arbor, MI, USA) was dissolved in DMSO and used at final concentration of 20 µM. Resveratrol (RV, cod. R5010; Sigma Aldrich) was dissolved in DMSO and used at final concentration of 100 µM. Cyclopamine hydrate (CP, cod. C4116; Sigma Aldrich) was dissolved in DMSO and used at final concentration of 5 µM. GANT61 (cod. G9048; Sigma Aldrich) was dissolved in DMSO and used at final concentration of 10 µM. Oxaliplatin (OxPt, cod. 13106; Cayman Chemicals) was dissolved in sterile water and used at final concentration of 50 µM. Control experiments demonstrated that DMSO (final concentration 0.01%) had no effect on cell growth, cell migration and autophagy. 

### 2.3. Antibodies

The following primary antibodies were employed for either immunofluorescence or Western blotting: mouse anti-LC3 (1:100, cod. 0231-100; Nanotools, Teningen, Germany), rabbit anti-LC3 (1:1000, cod. L7543; Sigma Aldrich), rabbit anti-TWIST1 (1:1000, cod. T6451; Sigma Aldrich), mouse anti-E-cadherin (1:50, cod. 610404; BD Biosciences, Franklin Lakes, NJ, USA), mouse anti-N-cadherin (1:50, cod. 610920; BD Biosciences), rabbit anti-BMI-1 (1:10,000 for Western blotting and 1:1000 for immunofluorescence, cod. ab126783; Abcam, Cambridge, UK), rabbit anti-GLI1 (1:1000, cod. PA5-32206; Thermo Fisher Scientific, Waltham, MA, USA), mouse anti-SNAIL (1:500, cod. 3895; Cell Signaling Technologies, Danvers, MA, USA), rabbit anti-BAX (1:500, cod. 2772: Cell Signaling Technologies), mouse anti-β-actin (1:2000, cod. A5441; Sigma Aldrich) and mouse anti-β-tubulin (1:1000, cod. T5201; Sigma Aldrich). The following were used as secondary antibodies for Western blotting: horse radish peroxidase (HRP)-conjugated goat anti-mouse (1:10,000; cod.170-6516, BioRad, Hercules, CA, USA) or goat anti-rabbit (1:10,000; cod.170-6515, BioRad). The followings secondary antibodies were used for immunofluorescence: AlexaFluor488-conjugated goat-anti-rabbit IgG antibody (1:1000; cod. A32731, Thermo Fisher Scientific) or AlexaFluor555-conjugated goat-anti-mouse IgG antibody (1:1000; cod. A32727, Thermo Fisher Scientific).

### 2.4. Wound-Healing Migration Assay

Cells were seeded in Petri dishes at a density of 50,000 cells/cm^2^ and cultured until 80–90% confluence. The cell monolayer was scratched with a sterile pipette yellow tip to produce a straight line, and the debris washed out with culture medium. To prevent starvation induction of autophagy, medium and treatments were renovated every 24 h. The open gap was photographed with a phase contrast microscope (magnification 5×, Zeiss AXIOVERT 40CFL, Oberkochen, Germany) at the indicated times. The rate of healing was estimated by ImageJ software based on the area free of cells, as previously reported [11]. Data are calculated for three different fields per each condition and represent three separate experiments.

### 2.5. Transwell In Vitro Cell Migration Assay

Cells were seeded in Petri dishes at a density of 25,000 cells/cm^2^ and cultured until reaching 80% confluence. Cell monolayers were cultured as appropriate, and treatments were renovated the following day. After 48 h of culture, cells were trypsinized, collected and counted. For each experimental condition, 50,000 cells were resuspended in serum-free medium supplemented with corresponding treatment and loaded into individual uncoated inserts containing 8.0 µM pore-size polycarbonate membrane (cod. 3422; Corning Incorporated Costar, New York, NY, USA). Each insert was placed in a 24-well plate containing complete media (10% FBS) and the plate was placed in the incubator. After 24 h (collectively 72 h of treatment), cells that had migrated to the underside of the inserts were washed in PBS, fixed in methanol for 30 min, washed in PBS and then stained for 1 h with eosin-hematoxylin solution (cod. 05-M06002; Bio-Optica, Milan, Italy). Inserts were washed twice in water and left to dry for 2–3 days. Inserts were cut and mounted onto slides using Biomount reagent (cod. 05-BMHM100; Bio-Optica) and photographed in random fields using a bright-field microscope (magnification 20×; Panoramic Midi, Sysmex). The number of migrated cells was estimated by ImageJ software using the auto-count function. Data were calculated for five different fields per each condition and represent three separate experiments.

### 2.6. One Color Microarray Genome-Wide Gene Expression Analysis

Cells were plated on Petri dishes at a density of 50,000 cells/cm^2^ and cultured until 70–80% confluence. Once they reached appropriate confluence, cell monolayers were treated with 20 µM LPA or 100 µM RV for 72 h. Medium and treatments were renovated every 24 h. Total RNA was isolated from the cells using an Absolutely RNA mRNA kit (Agilent Technologies, Palo Alto, CA, USA). mRNA was amplified and labeled by Amino Allyl MessageAmp II aRNA Kit (Ambion, Austin, TX, USA) using NHS ester Cy3 dye (Amersham Biosciences, Arlington Heights, IL, USA). Total RNA quality and labeling was checked by means of RNA 6000 Nanochip assays and run on the Agilent 2100 Bioanalyzer (Agilent Technologies, Santa Clara, CA, USA). Total RNA amplified and labeled mRNA concentrations were calculated using the NanoDrop ND-1000 Spectrophotometer (NanoDrop Technologies, Wilmington, DE, USA). Equal amounts (0.2 mg) of labeled specimens were fragmented and hybridized to Human Whole Genome Oligo Microarrays 860 K v2 (Agilent Technologies), representing 27958 Entrez Gene RNAs and 7419 lincRNAs. Each step was performed using the In-Situ Hybridization Kit-Plus (Agilent Technologies) and following the 60-mer oligo microarray processing protocol. Slides were then washed with the SSPE wash procedure and scanned using an Agilent Scanner version C (G2505C, Agilent Technologies). Images were analyzed using the Feature Extraction software v10.7. Raw data elaboration was carried out with Bioconductor (www.bioconductor.org, accessed on 1 March 2019), using R statistical language. Background correction was performed with the normal exponential (norm-exp.) method, and quantile was used for between-array normalization. The Linear Models for Microarray Analysis (LIMMA) package was then used to identify differentially expressed genes between the different experimental conditions. The empirical Bayes method was used to compute moderated t-statistics. Transcripts with a log base twofold change (logFC) greater than 0.20 or lower than −0.20 were considered as differentially expressed.

### 2.7. Bioinformatic Analysis

TBtools software (https://github.com/CJ-Chen/TBtools/, accessed on 8 September 2021) was used to identify the differentially expressed genes (DEGs) in LPA- or RV-treated cells that are represented in the form of Volcano plot. A *p*-value of ≤0.01 (*p*-value threshold was fixed above 2.0) and |log2 fold change| ≥ 1 were used as the cut-off criteria for this study.

DAVID (Database for Annotation, Visualization and Integrated Discovery) Bioinformatics Resources 6.8 (https://david.ncifcrf.gov/, accessed on 30 August 2021) software was used for Gene Ontology (GO) analysis to obtain biological processes enrichment within differentially expressed genes in LPA- or RV-treated cells. Data are presented in bar graphs which display the number of transcripts belonging to each up- or downregulated pathway.

The heat map representing the transcriptomic analysis was created by using MeV4 Multiple Experiment Viewer (http://mev.tm4.org/, accessed on 30 August 2021). For each experimental condition, the analysis was performed in duplicate, and data are shown in heat map double columns.

Kaplan–Meier curves, correlation studies and platinum response analysis were conducted by extracting clinical data from the TCGA database (www.portal.gdc.cancer.gov/, accessed on 6 May 2021). The analysis was conducted on an ovarian serous cystadenocarcinoma dataset (TCGA Nature 2011, that comprised 316 patients). mRNA expression and clinical information (including overall survival, disease-free status and platinum sensitivity/resistance) were downloaded from the cBioportal.org. Patients were grouped based on the level of mRNA expression. Low *versus* high groups were defined relative to the median expression level of overall patient cohort.

The correlation between the mRNA expression of relevant biomarkers and the response to platinum therapy is represented in histograms. These graphs report the number of patients with a specific transcriptome profile that were classified as resistant (disease progression within six months from primary therapy), sensitive (relapse or disease progression at least six months after the end of treatment) or too-early resistant (resistance occurs as soon as treatment start) on the basis of the clinical outcome reported in the database.

Survival curves reported in the Supplementary Figure S1 were obtained by using the Kaplan–Meier plotter (www./kmplot.com/, accessed on 15 December 2020) and the analysis was conducted on a larger ovarian patients’ dataset (that comprised 1656 samples) to corroborate the findings achieved with TCGA interrogation.

Scatter plots were employed to represent the correlation between the expression of relevant biomarkers in the patient cohort (Supplementary Figure S2). Regression was estimated by calculating Pearson’s correlation coefficients (r) and the relative *p*-values.

Statistical analyses were performed using R (3.6.1 version, The R Foundation for Statistical Computing, Vienna, Austria) and SAS software (9.4. version, SAS Institute Inc., Cary, NC, USA). The log-rank test has been used to determine the statistical significance. The *p*-value ≤ 0.05 was considered as significant.

### 2.8. Western Blot Analysis

Cells were seeded in Petri dishes at a density of 50,000 cells/cm^2^ and treated when confluence reached approximately 80%. Cell homogenates were prepared by freeze-thawing and ultrasonication in a lysis buffer containing protease inhibitors. Lysates were separated by SDS-PAGE and then transferred onto a PVDF membrane (cod. 162-0177; BioRad, Hercules, CA, USA). The membranes were blocked with 5% non-fat dry milk (cod. sc-2325; Santa Cruz Biotechnology, Santa Cruz, CA, USA) + 0.2% Tween-20 for 1 h at room temperature. Membranes were incubated with the specific primary antibody overnight at 4 °C, followed by incubation for 1 h at room temperature with the secondary HRP-conjugated antibody. The bands were detected using Enhanced Chemiluminescence reagents (ECL, cod. NEL105001EA; Perkin Elmer, Waltham, MA, USA) and developed using the ChemiDoc XRS instrument (BioRad). Intensity of the bands was estimated by densitometry using Quantity One Software (BioRad). Experiments were reproduced at least three times separately.

### 2.9. Immunofluorescence

Cells were plated on sterile coverslips, scratched, treated as indicated, and coverslips were processed for immunofluorescence. After cell fixation with ice-cold methanol and permeabilization with 0.2% Triton-PBS, the coverslips were incubated overnight at 4 °C with specific primary antibodies (diluted in 0.1% Triton-PBS + 10% FBS) and thereafter for 1 h at room temperature with secondary antibodies (diluted in 0.1% Triton-PBS + 10% FBS). Nuclei were stained with the UV fluorescent dye DAPI (4′,6-diamidino-2-phenylindole). Coverslips were mounted onto glasses using SlowFade reagent (cod. S36936; Life Technologies, Paisley, UK) and the wound areas were imaged under a fluorescence microscope (Leica DMI6000, Leica Microsystems, Wetzlar, Germany).

### 2.10. 3D Spheroids Forming Assay

Petri dishes were coated with 5 mg/mL poly-HEMA solution (cod. P3932; Sigma Aldrich) and left under the biological hood to completely dry, and then stored at room temperature until used. Cells were seeded at a density of 500,000 cells/Petri and treated depending on the experimental conditions. The growth of spheroids was monitored by taking pictures using a phase contrast microscope (magnification 20×, Zeiss AXIOVERT 40CFL) at each time point. The quantification of the dimension of the spheroids was performed using ImageJ software. The spheroids area was calculated for ten different fields per each condition and represents three separate experiments; 3D spheroid cultures were processed by cell imaging. Immunofluorescence was performed on spheroids cytospotted on glass slides. The latter were fixed in methanol, permeabilized for 30 min in 0.5% Triton-PBS and then processed as described in the immunofluorescence section.

### 2.11. Assessment of Autophagy in Living Cells Expressing GFP-LC3

SKOV3-GFP-LC3 cells were seeded on coverslips at a density of 25,000 cells/cm^2^ and cultured till confluence. A scratch-wound was made with a sterile pipette tip on each coverslip, and cell debris was washed out. The coverslips were then treated depending on the experiment performed. The wound area was photographed under the fluorescence microscope (Leica DMI6000, Leica Microsystems).

### 2.12. Gene Silencing

Post-transcriptional silencing was achieved by the small interference RNA (siRNA) technology. Cells were transfected with siRNA *BMI-1* or siRNA scrambled using Lipofectamine 3000 Reagent (cod. L3000-015, Life Technologies) as indicated by the purchaser. Cells were plated in a Petri dish or onto coverslips, depending on the experiment performed, at a density of 25,000 cells/cm^2^, and allowed to adhere 24 h before transfection. The siRNA-loaded liposomal complexes were prepared in Opti-MEM I Reduced Serum Medium (cod. 11058021, Life Technologies) with 100 pmol siRNA *BMI-1* or siRNA scrambled and 7.5 µL of Lipofectamine 3000. After 6 h of incubation, media was replaced with complete serum-containing culture medium (10% FBS), and the cells were cultivated for a further 24 h to allow the maximal gene silencing prior to any treatment. Thereafter, cells were treated depending on the experiment performed. Cell homogenates and coverslips were processed as reported above.

### 2.13. Imaging Acquisition and Analysis

Fluorescence images were acquired using the fluorescence microscope (Leica Microsystems, Wetzlar, Germany; DMI6000). For each experimental condition, at least three slides were prepared in separate experiments and five to ten microscopic fields randomly chosen were imaged by two independent investigators unaware of the treatment. Quantification of fluorescence intensity was performed with the software ImageJ. Representative images of selected fields are shown.

### 2.14. Statistical Analysis

Statistical analysis was performed using GraphPad Prism 5.0 software. Bonferroni’s multiple comparison test after one-way ANOVA analysis (unpaired, two-tailed) was employed. Significance was considered as follow: **** *p* < 0.0001; *** *p* < 0.001; ** *p* < 0.01; * *p* < 0.05. All experiments have been reproduced at least three times in separate and independent replicates. All data are reported as average ± S.D.

## 3. Results

### 3.1. Resveratrol Counteracts LPA-Induced Ovarian Cancer Cell Migration

We performed a wound-healing scratch assay in three ovarian cancer cell lines, SKOV3, OVCAR3 and OAW42, that differ in their genetic background and the grade of malignancy (details are reported in Supplementary Table S1), to determine the pro-migratory effect of LPA and the ability of resveratrol (RV) to contrast such effect. Cancer cells were treated with 20 µM LPA, 100 µM RV or both for 72 h; the culture media were renovated every 24 h to avoid secondary effects of starvation on cell migration. LPA accelerated cell migration in all three ovarian cancer cell lines at each time point considered when compared to the rate of healing in control condition, while RV inhibited ovarian cancer cell motility, reducing the rate of healing by approximatively 60–70% compared to untreated cells. This effect was evident even in the presence of LPA, and by 72 h only 40% of the wound was healed in the presence of RV. Similar trends were observed in all the three cell models, indicating that RV opposes LPA-induced motility of ovarian cancer cells bearing different genetic alterations.

### 3.2. Resveratrol and LPA Oppositely Modulate the Transcriptomic of Ovarian Cancer Cells and Particularly the Genes of the Hedgehog Pathway and Epithelial-to-Mesenchymal Transition

**Figure 1:**
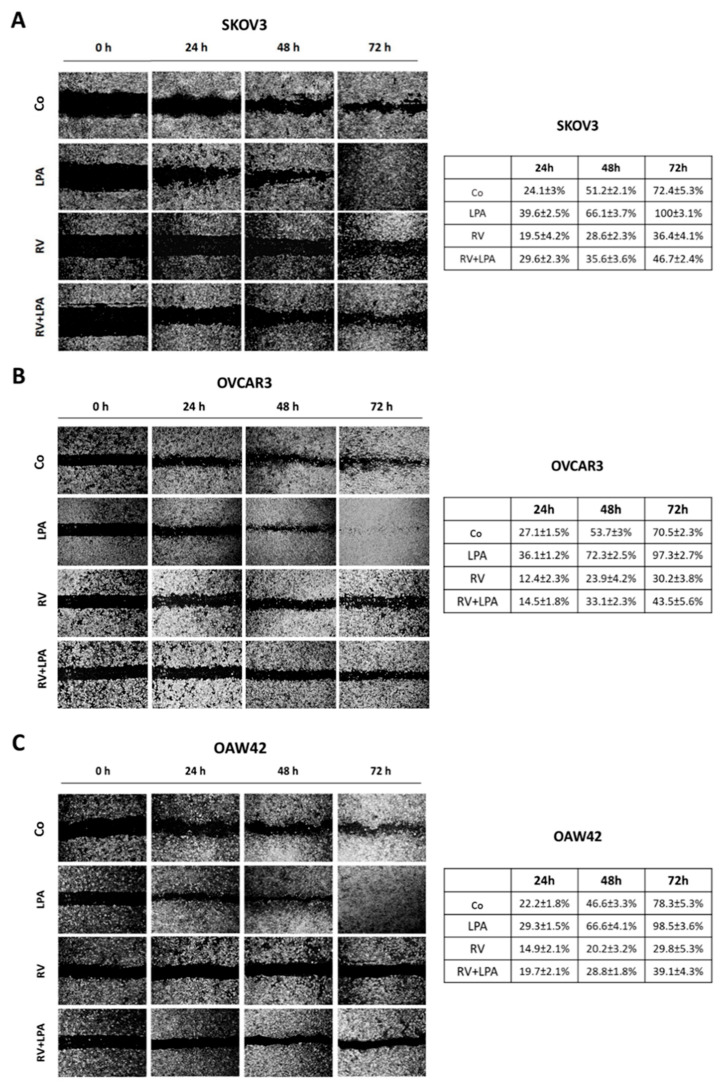
Resveratrol counteracts LPA-induced cell migration in three different ovarian cancer cell models. SKOV3 (**A**), OVCAR3 (**B**) and OAW42 (**C**) human ovarian cancer cells were plated on Petri dishes and let grow to confluence. Cell monolayers were scratched to produce a straight line. Cells were exposed to 100 µM resveratrol (RV) or to 20 µM LPA or both simultaneously. Medium was replaced and substances re-added every 24 h. Phase-contrast photos of the open gap were taken at time points 0, 24, 48 and 72 h. Table reports the rate of healing (%) for each time point estimated using ImageJ software. Data are presented as the average ± S.D. calculated for three different fields per each condition in three separate experiments.

In searching for the molecular pathways underlying the opposite effects of LPA and RV, we performed a whole genome expression profiling of ovarian cancer cells exposed to RV or LPA for 72 h. SKOV3 cells were chosen as representative because of their apparent highest response to LPA pro-migratory stimulation. We selected the 72 h time point since we then observed the major phenotypic changes in terms of cell migration (Figure 1A).

**Figure 2:**
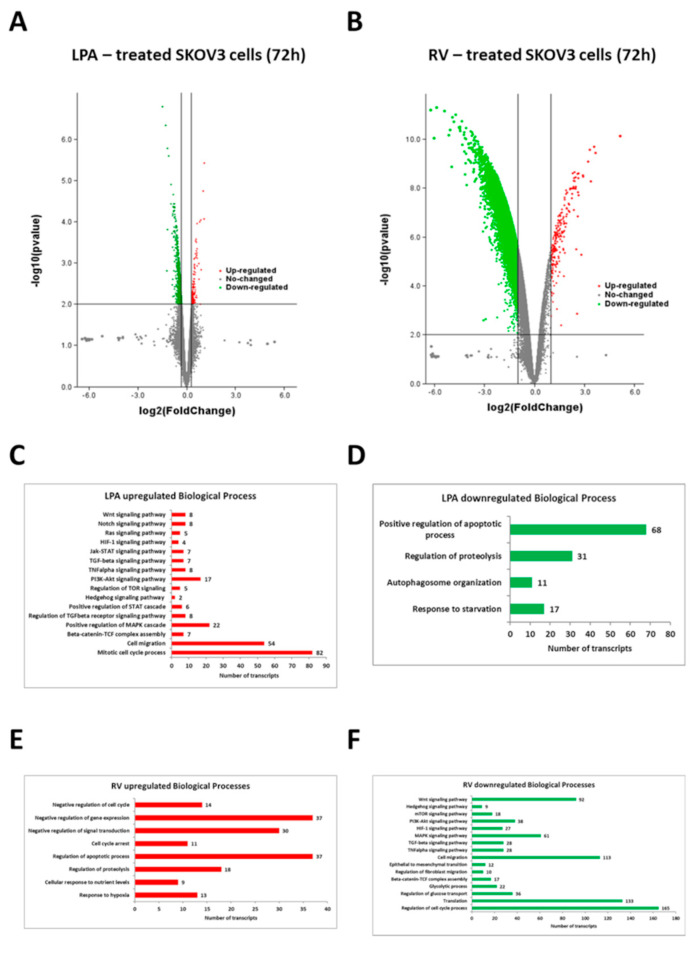
Differential modulation of biological processes by resveratrol and LPA in SKOV3 ovarian cancer cells. (**A**) Volcano plot displaying the differential expressed genes (DEGs) in LPA-treated cells. Red dots represent DEGs with log2 (fold change) value > 1.0, while green dots represent the DEGs with log2 (fold change) value < −1.0. (**B**) Volcano plot displaying the differential expressed genes (DEGs) in resveratrol (RV)-treated cells. Red dots represent DEGs with log2 (fold change) value > 1.0, while green dots represent the DEGs with log2 (fold change) value < −1.0. (**C**) Graph reporting LPA upregulated biological processes. (**D**) Graph reporting LPA downregulated biological processes. (**E**) Graph reporting RV upregulated biological processes. (**F**) Graph reporting RV downregulated biological processes.

When comparing the gene sets with significant enrichment represented as Volcano plots of RV *versus* LPA (Figure 2A,B), it appears evident that RV modulated a higher number of transcripts than LPA. Regarding the biological processes mainly subjected to modulation, LPA upregulated the transcripts of oncogenic pathways supporting stemness, cell survival, cell proliferation (including the mitotic cell cycle process) and migration while it downregulated the genes associated with apoptosis, proteolysis and autophagy processes (Figure 2C,D); on the other hand, RV markedly increased the expression of genes associated with cell cycle arrest, apoptosis and proteolysis while it strongly downregulated the transcripts involved in the invasive behavior of ovarian cancer cells such as EMT-promoting factors, pro-angiogenic molecules, glycolysis, cell cycle progression and cytokine-triggered pathways (Figure 2E,F).

**Figure 3:**
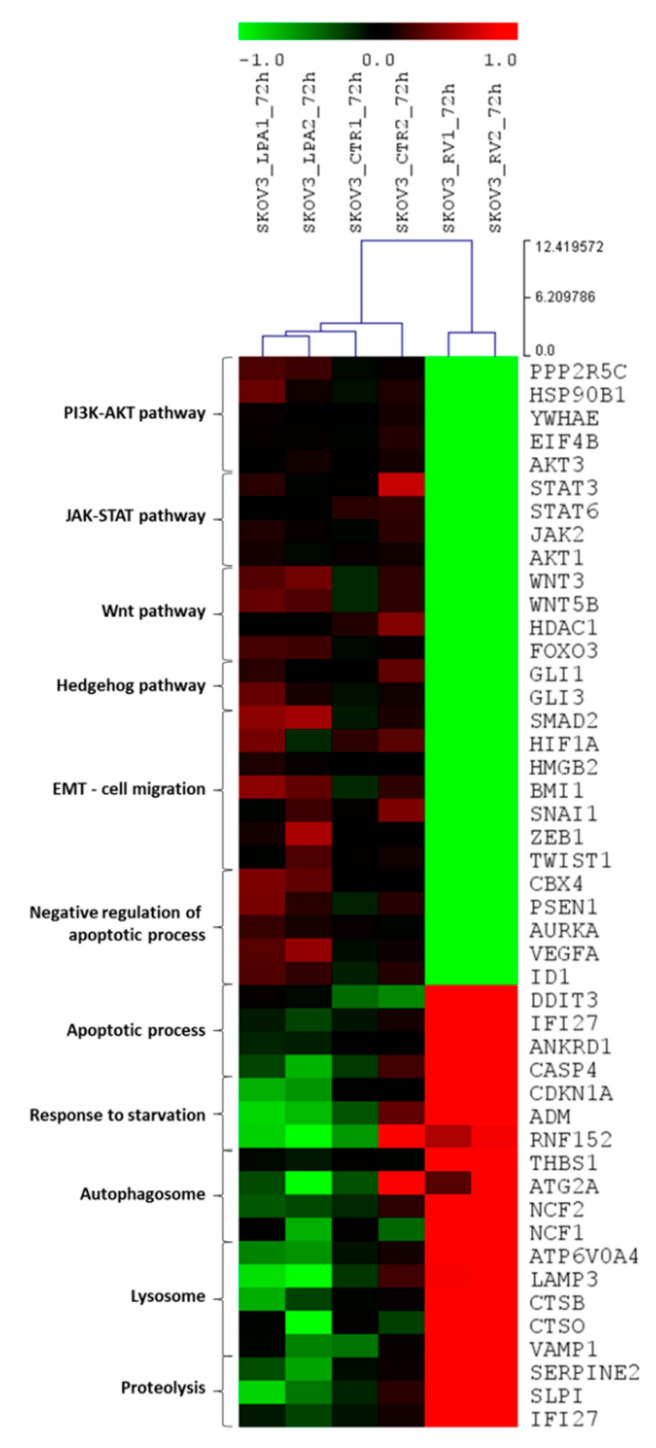
Resveratrol downregulates the transcriptome associated with the Hedgehog/EMT pathway while upregulating autophagy-related genes. Heat map of the expression profiles of the top differentially modulated genes belonging to selected pathways regulating cancer cell locomotion and chemoresistance. Transcriptome of SKOV3 cells upon LPA treatment (first and second column) or RV treatment (fifth and sixth column) is compared to the expression signature of untreated cells (third and fourth column). Green and red colors represent downregulation and upregulation, respectively.

Next, we focused on the genes involved in the biological processes associated with major malignant features, namely stemness, cell proliferation, cell migration, resistance to cell death and dysregulated autophagy. The top significant differentially regulated genes by RV and LPA related to these processes are shown in the heat map reported in Figure 3. It appears evident that LPA upregulated and RV downregulated a range of transcripts of the PI3K-AKT, Wnt, Notch, JAK-STAT, mTOR, Hh and TGFβ pathways and of EMT process, while RV upregulated and LPA downregulated the transcripts for apoptosis and autophagy-lysosomal proteolysis (Figure 3).

### 3.3. Resveratrol Reverses LPA-Induced EMT along with Restoration of Autophagy

In our previous studies we found that RV could revert the pro-migratory effect of stromal IL-6 on ovarian cancer and cholangiocarcinoma cells by rescuing autophagy from inhibition [11,12]. Thus, we asked whether RV was acting in the same way for counteracting LPA.

**Figure 4:**
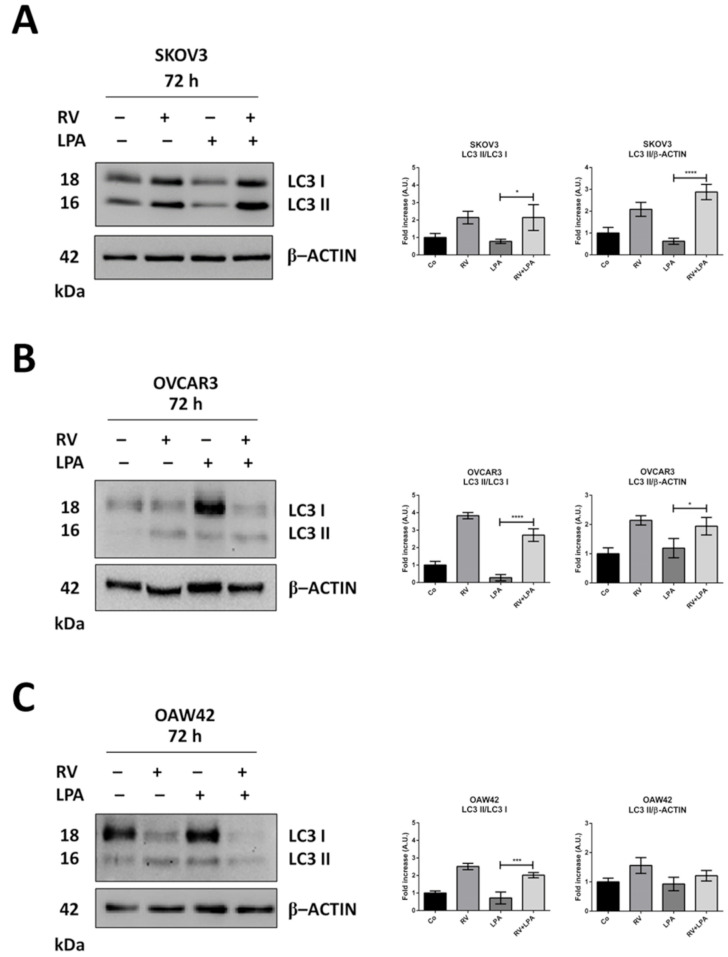
Resveratrol restores autophagy flux inhibition by LPA. SKOV3 (**A**), OVCAR3 (**B**) and OAW42 (**C**) human ovarian cancer cells were plated on Petri dishes and let grow to confluence. Cells were exposed for 72 h to 100 µM resveratrol (RV) or 20 µM LPA or both simultaneously. Cell homogenates were analyzed by Western blotting for the expression of LC3. Autophagy flux was monitored by conversion rate of the precursor LC3-I to the lipidated mature form LC3-II, whereas the autophagosome accumulation was monitored by LC3-II/β-actin ratio. All blots are representative of three independent experiments. Densitometric data ± S.D. representing three replicates are reported in the graphs. Statistical analysis was performed by using GraphPad Prism 5.0 software. Bonferroni’s multiple comparison test after one-way ANOVA analysis (unpaired, two-tailed) was employed. Significance was considered as follow: **** *p* < 0.0001; *** *p* < 0.001; * *p* < 0.05.

First, the effects of LPA and RV on autophagy regulation were assessed by determining by Western blotting the rate of LC3-II formation and accumulation in the cells (Figure 4). LPA prevented the conversion of LC3-I (cytoplasmic form) into LC3-II (autophagosomal form) and the accumulation of the latter, indicating the inhibition of autophagy; on the other hand, RV confirmed its ability to enhance autophagy and to restore it even in the presence of LPA. These effects were more evident in SKOV3 cells that were *TP53* null compared to OAW42 cells that expressed wild-type *TP53*.

**Figure 5:**
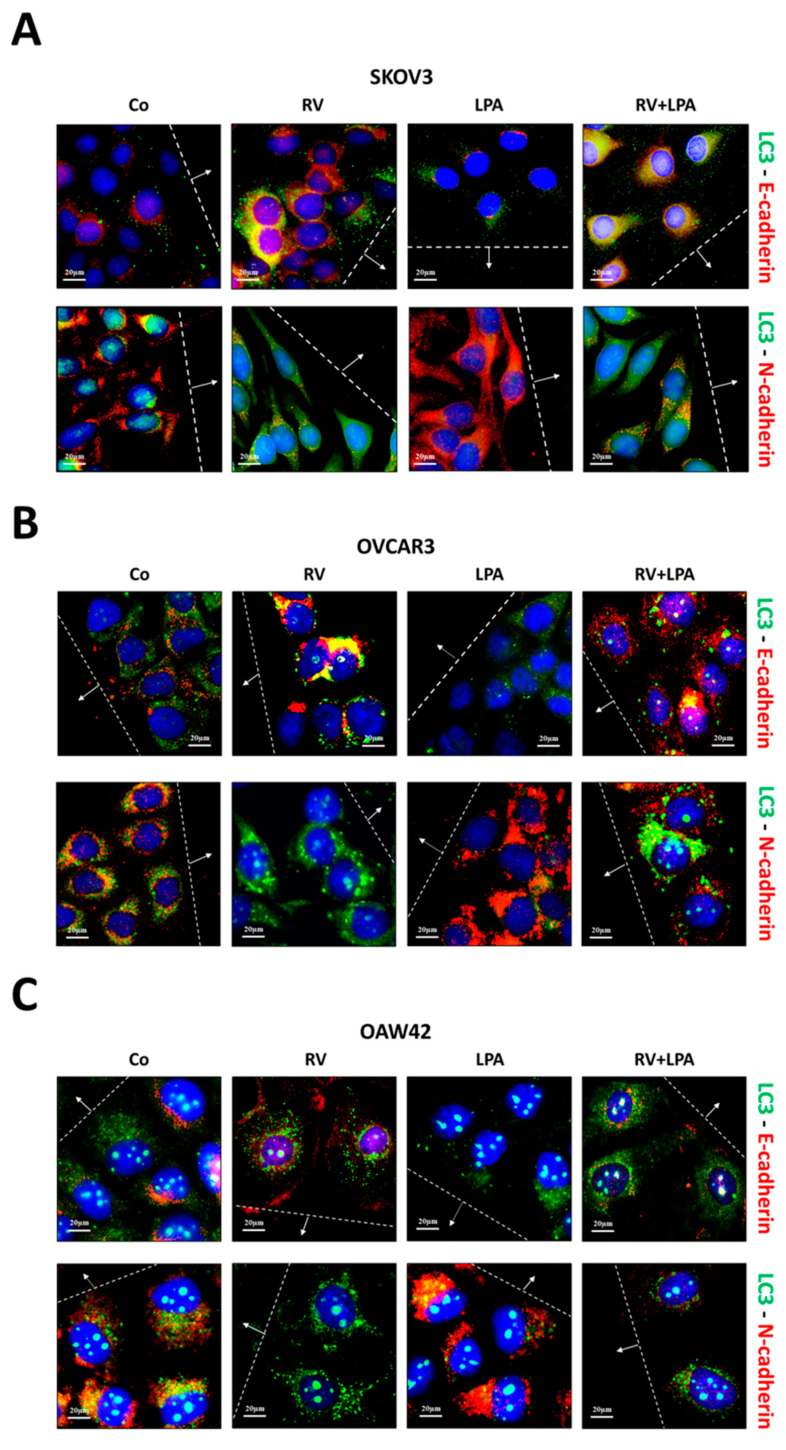
Resveratrol reverts LPA-mediated EMT in parallel with autophagy induction. SKOV3 (**A**), OVCAR3 (**B**) and OAW42 (**C**) human ovarian cancer cells were plated on coverslips, let grow and scratched using a pipette tip. Cells were exposed for 72 h to 100 µM resveratrol (RV) or 20 µM LPA or both simultaneously. Coverslips were fixed and two different staining were performed: LC3 (red) and E-cadherin (green) and LC3 (red) and N-cadherin (green). All experiments were reproduced three times. Images were taken in the proximity of the migration front. Scale bar = 20 µm; magnification = 63×.

Next, we detected by immunofluorescence co-staining LC3 (autophagosome marker) and E-cadherin or N-cadherin (EMT markers) in the cells at the migration front. As shown in Figure 5, LPA-treated cells displayed a mesenchymal switch from E-cadherin to N-cadherin along with LC3 downregulation, while the treatment with RV reverted the EMT phenotype in the cells near the wound, which showed a marked increment of LC3-positive puncta even in the presence of LPA. Taken together, these data support the view that the pro-migratory effect induced by LPA associates with the inhibition of autophagy in the cells.

### 3.4. LPA Stimulates While Resveratrol Inhibits the Hedgehog-Mediated EMT

**Figure 6:**
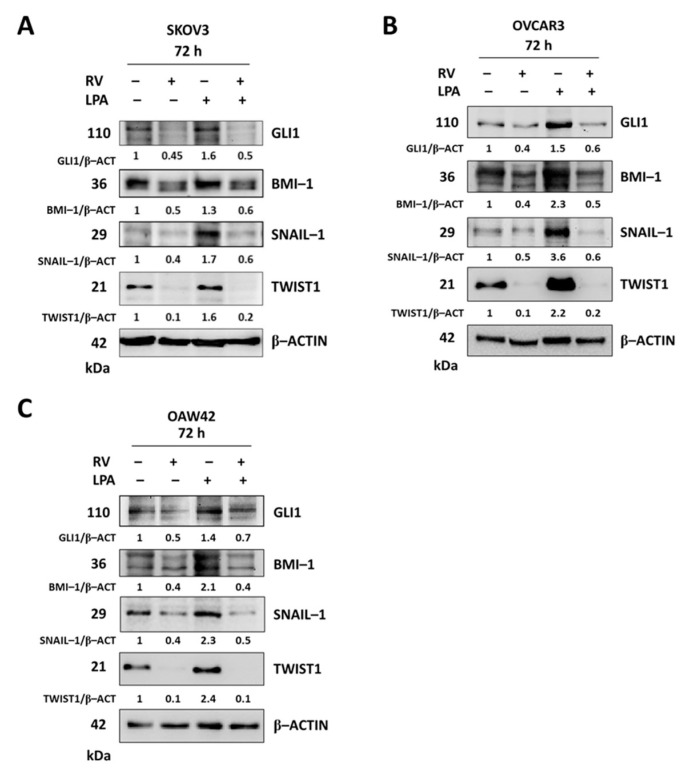
Resveratrol attenuates the upregulation of Hedgehog/EMT signaling induced by LPA. SKOV3 (**A**), OVCAR3 (**B**) and OAW42 (**C**) human ovarian cancer cells were plated on Petri dishes and let grow to confluence. Cells were exposed for 72 h to 100 µM resveratrol (RV) or 20 µM LPA or both simultaneously. Cell homogenates were analyzed by Western blotting for the expression of GLI1, BMI-1, SNAIL-1 and TWIST1. The membranes were stripped and re-probed with different antibodies. The β-actin for OVCAR3 and OAW42 correspond to the ones reported in Figure 4. Densitometric data (normalized on the loading control β-actin) are reported. All blots are representative of three independent experiments.

EMT in ovarian cancer cells has been attributed to the aberrant activation of the Hedgehog (Hh) pathway [25,26]. In our experimental models the transcripts of the TGFβ-Hh-TWIST pathways were among those differently modulated by LPA and RV (Figure 2). Therefore, we validated at protein level the opposite modulation of the main activators of the Hh pathway (GLI1) and of the EMT (BMI-1, SNAIL-1 and TWIST1) process, as emerged from the transcriptomic analysis. In all three cell lines tested, LPA upregulated GLI1 (that in turn activates Hh signaling) and increased the expression of the three EMT activators, while RV markedly downregulated the expression of GLI1 and of the EMT activators. RV effects were still evident even in the presence of LPA, suggesting that under this condition the LPA-induced EMT program in ovarian cancer cells was hampered through Hh downmodulation (Figure 6).

To validate this interpretation, we tested the ability of two different Hedgehog inhibitors, namely cyclopamine (CP), that antagonizes Smoothened (Smo) activity, and GANT61, that specifically targets GLI1-mediated transcription, to inhibit LPA-induced ovarian cancer cell migration (SKOV3 cells were chosen as representative).

**Figure 7:**
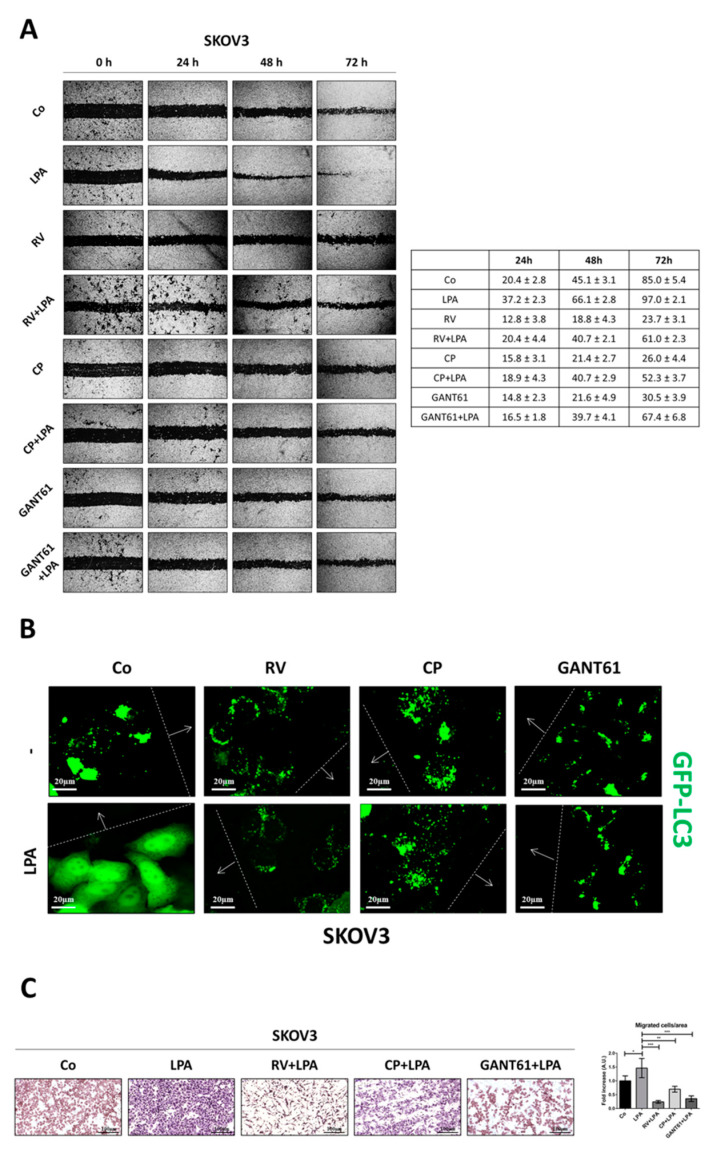
Resveratrol hampers LPA-induced Hedgehog-mediated cell motility while upregulating autophagy in the cells at the migration front. (**A**) A wound was made in SKOV3 cultured to confluency in Petri dishes. The cells were then exposed to 100 µM resveratrol (RV), 5 µM cyclopamine (CP) or 10 µM GANT61 in the presence/absence of 20 µM LPA. Medium was replaced and substances re-added every 24 h. Phase-contrast photos of the open gap were taken at time points 0, 24, 48 and 72 h. Graph reporting the rate of healing (%) for each time point estimated using ImageJ software. Data represent the average ± S.D. calculated for three different fields per each condition in three separate experiments. (**B**) SKOV3-GFP-LC3 cells were cultured to confluency on coverslips, then wounded and treated as in panel A. At the end of treatment, coverslips were washed, mounted and immediately imaged under the fluorescence microscope. Images were taken in the proximity of the migration front. Scale bar = 20 µm; magnification = 63×. (**C**) SKOV3 cells were cultured in Petri dishes and treated as described in panel A. After 48 h, cells were collected and counted. An equal number of cells for each experimental condition was seeded in the upper chambers of a Transwell migration insert. The upper chamber contained serum-free media supplemented as indicated, whereas the below chambers were filled with complete media (+10% FBS) to produce a chemotactic gradient. After 24 h of culture, Transwell inserts were washed, fixed in methanol and stained with eosin-hematoxylin. Phase-contrast photos of the migrated cells are shown. Scale bar = 100 µm; magnification = 20×. Graph reporting the number of cells that had migrated as estimated by ImageJ software. Data represent the average ± S.D. calculated for three random fields per each condition in three separate experiments. Statistical analysis was performed by using GraphPad Prism 5.0 software. Bonferroni’s multiple comparison test after one-way ANOVA analysis (unpaired, two-tailed) was employed. Significance was considered as follow: *** *p* < 0.001; ** *p* < 0.01; * *p* < 0.05.

The wound-healing assay proved that CP and GANT61 markedly reduce the healing rate of ovarian cancer SKOV3 cells even under LPA stimulation, resembling the inhibitory effect of RV (Figure 7A). Notably, upon treatment with RV or Hh inhibitors the cells at the migration front display an autophagy induction even in the presence of LPA, as indicated by the formation of GFP-LC3-positive spots (Figure 7B).

The anti-migratory effects of these inhibitors were further tested in a Transwell migration assay. The amount of migrated cells, as assessed colorimetrically, increased under LPA stimulation by approximately 50% and it was greatly reduced by two-three fold in RV + LPA-, CP + LPA- and GANT61 + LPA-treated cells (Figure 7C).

### 3.5. BMI-1 Silencing Restores Autophagy and Prevents LPA-Induced EMT

**Figure 8:**
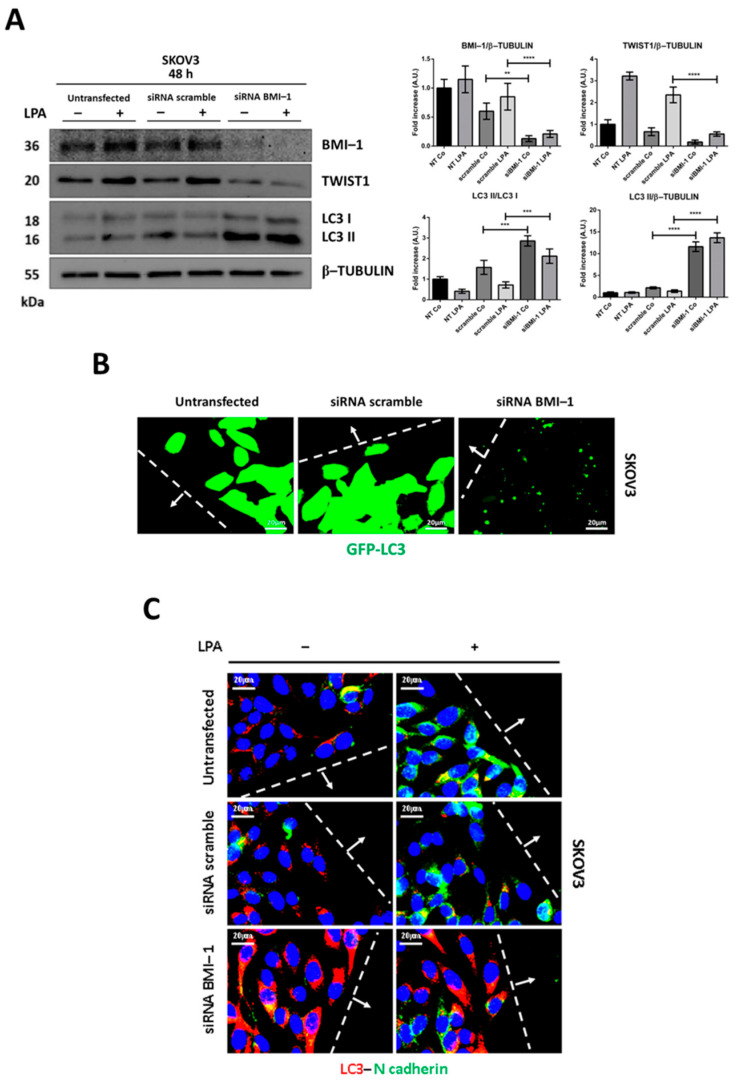
*BMI-1* knockdown results in downregulation of EMT in parallel with autophagy induction. (**A**) SKOV3 cells were plated on Petri dishes and let adhere. Once they reached the appropriated confluence, cells were transfected with siRNA *BMI-1* or siRNA scrambled and after 36 h from transfection, coverslips were incubated or not with 20 µM LPA. Medium was replaced and LPA re-added after 24 h. Cell homogenates were analyzed by Western blotting for the expression of LC3, TWIST1 and BMI-1. All blots are representative of three independent experiments. Densitometric data ± S.D. representing three replicates are reported in the graphs. Statistical analysis was performed by using GraphPad Prism 5.0 software. Bonferroni’s multiple comparison test after one-way ANOVA analysis (unpaired, two-tailed) was employed. Significance was considered as follow: **** *p* < 0.0001; *** *p* < 0.001; ** *p* < 0.01. (**B**) SKOV3-GFP-LC3 cells were plated on coverslips and let grow. Thereafter, cells were transfected with siRNA *BMI-1* or siRNA scrambled. The following day, coverslips were scratched with a yellow tip and cells were cultured for further 24 h. Coverslips were washed, mounted and immediately imaged under the fluorescence microscope. Imaging of GFP-LC3 fluorescence was performed in proximity of the migration front. Scale bar = 20 µm; magnification = 63×. (**C**) SKOV3 cells were plated on coverslips and let grow. Thereafter, cells were transfected with siRNA *BMI-1* or siRNA scrambled. The following day, coverslips were scratched with a yellow tip and cells were incubated or not with 20 µM LPA for 24 h. Coverslips were fixed and stained for LC3 (red)—N-cadherin (green). Images were taken in the proximity of the migration front. Scale bar = 20 µm; magnification = 63×.

BMI-1 (B-cell-specific Moloney murine leukemia virus integration site 1), a core member of the polycomb repressive complex 1 acting as an epigenetic silencer with oncogenic functions, has been shown to repress autophagy [27]. To confirm the role of Hh pathway in autophagy-dependent EMT as dictated by LPA or RV, we decided to genetically manipulate the expression of BMI-1. The latter is in fact downstream to the Hh pathway [28] and it is mechanistically linked to the TWIST1 pathway and EMT [29]. *BMI-1* silencing in SKOV3 cells led to an induction of autophagy (as indicated by LC3-II formation and accumulation) and in parallel to a reduction of TWIST1 expression (Figure 8A). Notably, no changes in LC3 and TWIST1 protein level were appreciable when LPA was added to *BMI-1*-knocked down cells. Additionally, *BMI-1*-silenced cells at the migration front displayed an increase of GFP-LC3-positive spots, while sham-transfected and untransfected cells exhibited diffuse GFP fluorescence, indicative of low basal autophagy (Figure 8B). Finally, *BMI-1*-silenced cells near the wound exhibited a downregulation of N-cadherin in parallel with an increase of LC3 levels even when exposed to LPA (Figure 8C). Taken together, the above data confirm that *BMI-1* knockdown rescues autophagy and inhibits EMT in ovarian cancer cells exposed to LPA resembling the effect of RV.

### 3.6. Ovarian Cancer Patients with Low Expression of Hedgehog/EMT Markers along with Upregulated Autophagy Have a Better Prognosis

Finally, we addressed whether the LPA-promoted signature associated with Hh (*GLI1*, *BMI1*) and EMT (*SNAI1*, *TWIST1*) genes has an impact on the clinical outcome of ovarian cancer patients. First, we interrogated a large dataset of ovarian cancer cases (n = 1656) from the Kaplan–Meier plotter. From the survival plots reported in Supplementary Figure S1, we found that *SNAI1* expression does not correlate with any significant change in the patients’ survival. Conversely, patients expressing low *GLI1* or low *TWIST1* had a longer overall survival than those belonging to the respective high expressing groups. Notably, patients bearing low expression of *BMI1* exhibited a significantly better overall survival (*p* = 5.6 ȕ 10^−5^) than those with high *BMI1* expression, indicating that this gene has a relevant prognostic value for the outcomes of ovarian cancer patients.

**Figure 9:**
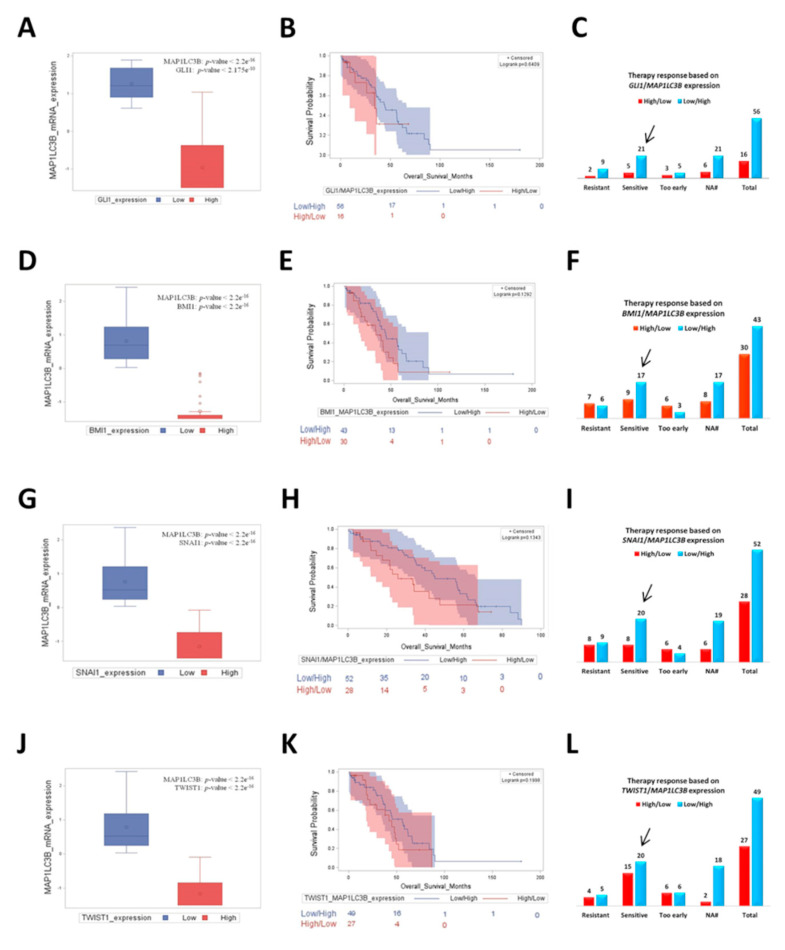
Patients with low expression of Hedgehog/EMT markers together with *MAP1LC3B* upregulation had a better overall survival and were more responsive to platinum therapy. We compared the *in vitro* responsiveness of cancer cells to oxaliplatin with the clinical outcomes of the ovarian cancer patients by interrogating the TCGA bioportal (ovarian serous cystadenocarcinoma dataset, TCGA Nature 2011). Those patients for whom therapy response status was not available are classified in N/A group. (**A**) Box plot showing the distribution of *MAP1LC3B* mRNA expression according to *GLI1* mRNA levels (high vs. low group). (**B**) Kaplan–Meier plot representing the overall survival status of patients stratified on the basis of the differential expression of *GLI1* and *MAP1LC3B* (high *GLI1*/low *MAP1LC3B* vs. low *GLI1*/high *MAP1LC3B*). (**C**) Graph showing the response to platinum therapy based on the differential mRNA expression of *GLI1* and *MAP1LC3B*. The histograms report the number of patients that were resistant, sensitive or developed chemoresistance as soon as the chemotherapy started (too-early group). (**D**) Box plot showing the distribution of *MAP1LC3B* mRNA expression according to *BMI1* mRNA levels (high vs. low group). (**E**) Kaplan–Meier plot representing the overall survival status of patients stratified in high *BMI1*/low *MAP1LC3B* vs. low *BMI1*/high *MAP1LC3B*. (**F**) Graph showing the response to platinum therapy based on the differential mRNA expression of *BMI1* and *MAP1LC3B*. (**G**) Box plot showing the distribution of *MAP1LC3B* mRNA expression according to *SNAI1* mRNA levels (high vs. low group). (**H**) Kaplan–Meier plot representing the overall survival status of patients stratified in high *SNAI1*/low *MAP1LC3B* vs. low *SNAI1*/high *MAP1LC3B*. (**I**) Graph showing the response to platinum therapy based on the differential mRNA expression of *SNAI1* and *MAP1LC3B*. (**J**) Box plot showing the distribution of *MAP1LC3B* mRNA expression according to *TWIST1* mRNA levels (high vs. low group). (**K**) Kaplan–Meier plot representing the overall survival status of patients stratified in high *TWIST1*/low *MAP1LC3B* vs. low *TWIST1*/high *MAP1LC3B*. (**L**) Graph showing the response to platinum therapy based on the differential mRNA expression of *TWIST1* and *MAP1LC3B*.

Next, we extended our study by performing a bioinformatic analysis on TCGA database to evaluate whether these findings correlate with autophagy modulation in patients’ samples. We compared the survival outcome of patients bearing differential opposite expression of Hh/EMT genes and of the autophagosomal marker MAP1LC3B and their responsiveness to platinum therapy (Figure 9). We found that the mRNA expression of *GLI1*, *BMI1*, *SNAI1* and *TWIST1* were inversely correlated with *MAP1LC3B* levels (Figure 9A,D,G,J), thus supporting the observations of the *in vitro* studies above. In addition, the scatter plots reported in the Supplementary Figure S2 confirmed these trends. Again, we observed that patients with low expression of Hh/EMT markers together with *MAP1LC3B* upregulation had a better outcome in terms of longer overall survival (Figure 9B,E,H,K) and of the response to platinum therapy. In fact, the numbers of patients that were sensitive to platinum treatment were higher compared to those ones recorded in the cohort of patients with high levels of Hh/EMT expression and exhibiting downregulation of autophagy (Figure 9C,F,I,L).

### 3.7. Resveratrol and Hedgehog Inhibitors Rescue Cell Growth Control and Responsiveness to Platinum

**Figure 10:**
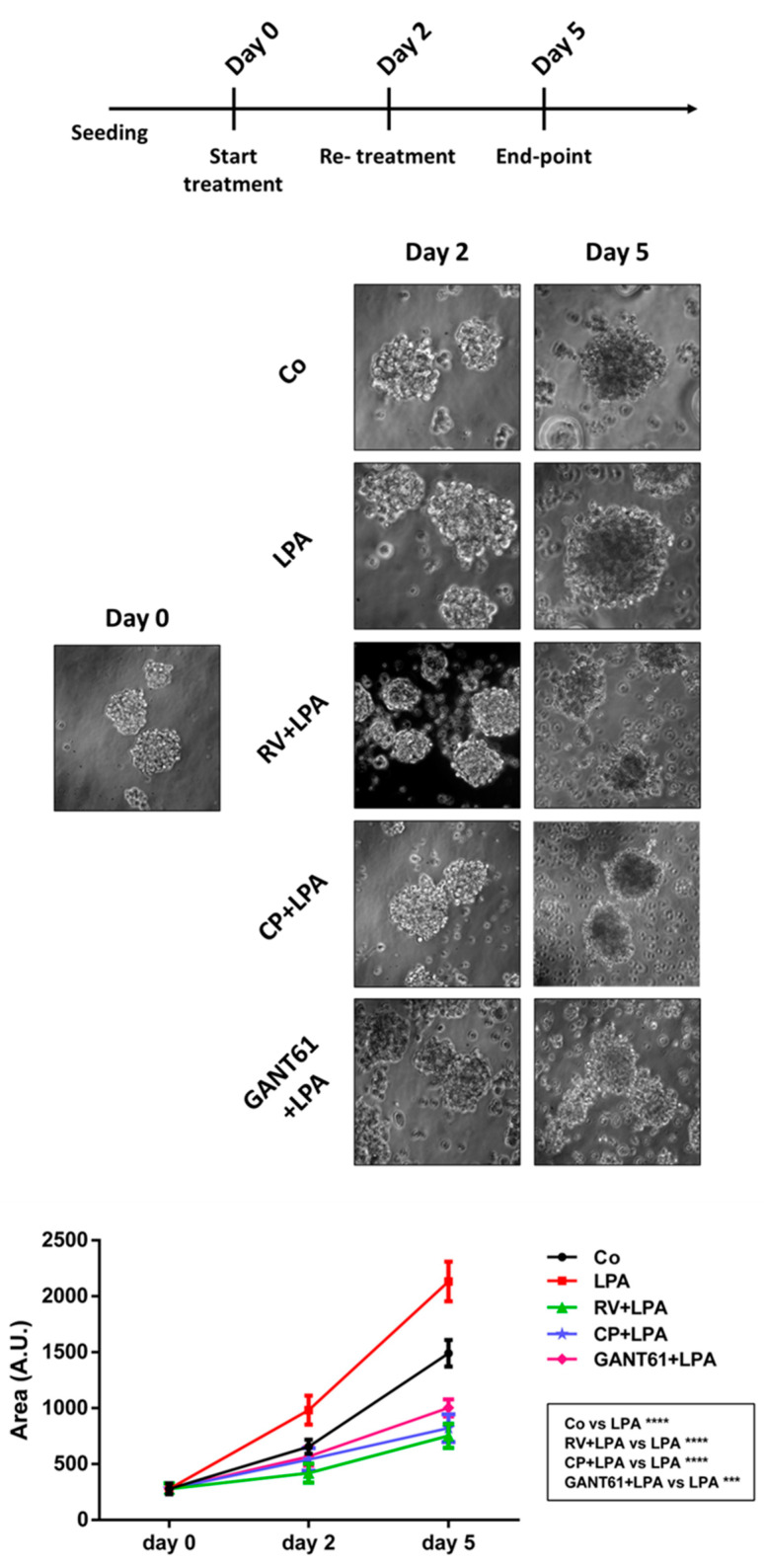
Resveratrol counteracts LPA-induced 3D ovarian cancer spheroids’ growth. SKOV3 cells were seeded on Poly-HEMA-coated Petri dishes and let grow until formation of 3D spheroids. At day 0, cells were exposed to 100 µM resveratrol (RV), 5 µM cyclopamine (CP) or 10 µM GANT61 in the presence/absence of 20 µM LPA. The experimental timeline is reported in the graphical scheme. The 3D spheroids were cultured for 5 days. Representative phase-contrast images of time-course experiment to monitor 3D spheroids’ growth. Magnification = 20×. The growth curve represents the area of 3D spheroids estimated at each time point. Statistical analysis was performed by using GraphPad Prism 5.0 software. Bonferroni’s multiple comparison test after one-way ANOVA analysis was employed. Significance was considered as follow: **** *p*< 0.0001; *** *p* < 0.001.

Finally, we investigated whether targeting the Hh-autophagy pathway with RV or Hh inhibitors could have therapeutic implication. The 3D spheroid model mimics the *in vivo* growth of ovarian cancer cells in the peritoneum [30,31], and therefore it was chosen for testing the ability of RV, CP and GANT61 to limit the pro-tumorigenic activity of LPA in terms of cancer cell growth and responsiveness to standard drug treatment. LPA promoted the proliferation of 3D spheroids, as determined by their dimension (the quantification of the area is reported in the growth curve), while RV, CP and GANT61 greatly limited the growth of such spheroids even when LPA was present (Figure 10).

**Figure 11:**
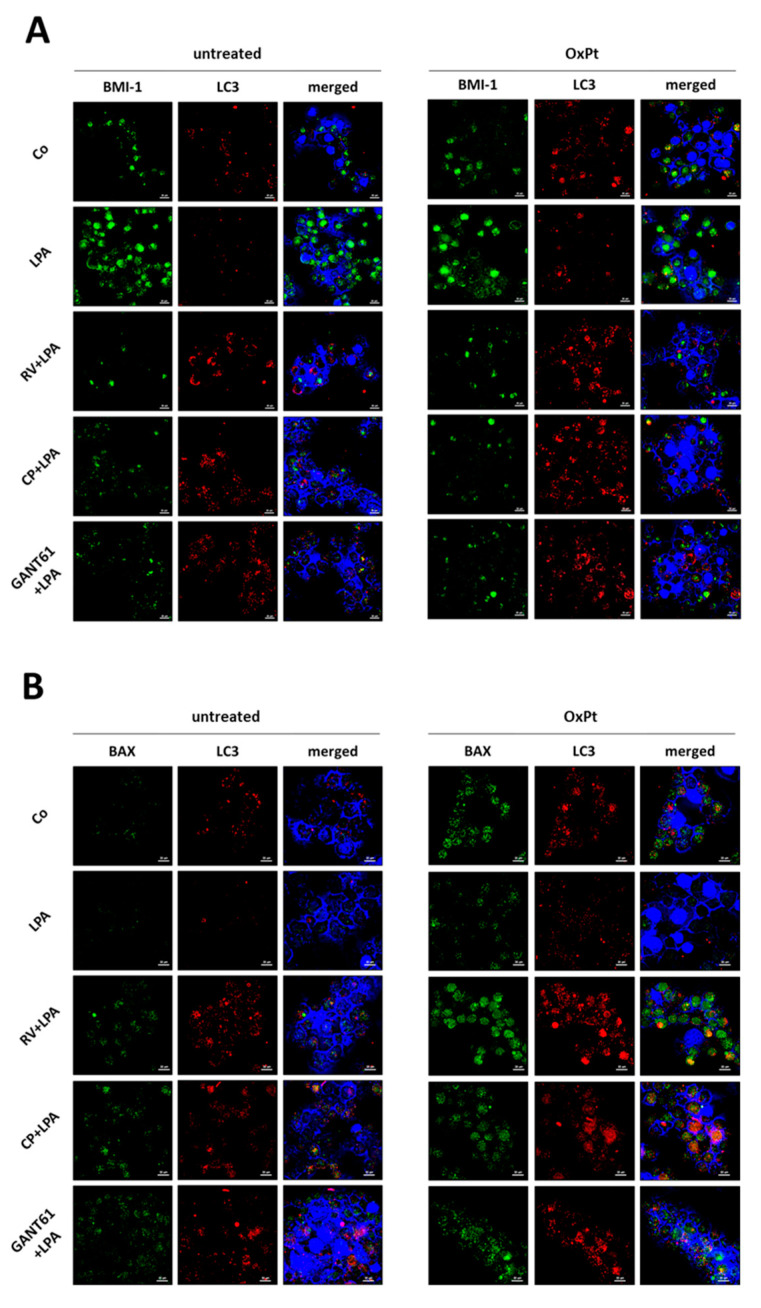
Resveratrol sensitizes LPA-treated 3D spheroids to chemotherapy. SKOV3 cells were seeded on Poly-HEMA-coated Petri dishes and let grow until formation of 3D spheroids. At day 0, cells were exposed to 100 µM resveratrol (RV), 5 µM cyclopamine (CP) or 10 µM GANT61 in the presence/absence of 20 µM LPA. 50 μM oxaliplatin (OxPt) was added at day 2. The 3D spheroids were cultured for 5 days, monitored with the phase-contrast microscope and photographed. Scale bar = 20 µm; magnification = 63×. (**A**,**B**) 3D spheroids collected at day 5 were cytospotted on glass slides. The slides were processed for immunofluorescence of BMI-1 (green)—LC3 (red) (panel **A**) and of BAX (green)—LC3 (red) (panel **B**).

Chemotherapy resistance in ovarian cancer has been associated with the abnormal activation of the Hh pathway [32,33,34]. Particularly, BMI-1, as a downstream effector of the Hh pathway [35], has been involved in acquisition of chemoresistance [36]. We therefore tested in the 3D spheroid model whether the responsiveness to oxaliplatin (OxPt) involved a crosstalk between the BMI-1-dependent autophagy and BAX-mediated apoptosis pathways. To this end, we performed the immunofluorescence double-staining of BMI-1-LC3 and of LC3-BAX in 3D spheroids exposed or not to OxPt in the absence or the presence of LPA along with RV or CP or GANT61. LPA-treated spheroids displayed high expression of BMI-1 in parallel with low levels of LC3 (as expected based on above data), and when treated with OxPt the level of these proteins was unchanged and BAX expression was negligible (Figure 11). Interestingly, in RV + LPA-treated spheroids we detected a marked decrease of BMI-1 expression along with the upregulation of LC3-positive puncta, and upon treatment with OxPt the expression of BAX increased in a large proportion of the cells that were positive also for LC3 (Figure 11). A similar trend was observed in the 3D spheroids exposed to the Hh inhibitors. Taken together, these data indicate that RV-induced BMI-1 downregulation leading to upregulation of autophagy sensitizes ovarian cancer cells to platinum therapy.

## 4. Discussion

The tumor microenvironment (TME) has been well-recognized as a pivotal player in favor of ovarian cancer malignancy. Its cellular components communicate with the cancer cells and, in turn, support the proliferation and metastasis by providing metabolites and releasing soluble factors that create a permissive niche for tumor expansion and invasion [5,37].

Lysophosphatidic acid (LPA) is a phospholipid growth mediator secreted by cancer cells and CAFs and is present in elevated concentrations in serum and ascites of ovarian cancer patients. LPA receptors (LPARs) are widely expressed in ovarian cancer tissues and the LPA/LPAR axis regulates many oncogenic processes by impinging on many targets involved in cell proliferation, survival, migration and invasion, angiogenesis, glycolytic and lipogenic metabolism, and inflammation [38,39].

Here we report novel pathways involved in LPA pro-tumorigenic activities as discovered through transcriptomic and bioinformatic analyses and mechanistically validated in *in vitro* 2D and 3D spheroid ovarian cancer models. The latter is assumed as a valuable mimic of the *in vivo* growth of ovarian cancer in the peritoneal cavity [30,31].

RV is a nutraceutical and caloric restriction mimetic with strong autophagy-inducing activity [10] and known anti-cancer properties in ovarian cancer [40,41]. RV has been shown to modulate the activity of the cells in the TME, attenuating the release of pro-tumorigenic and immune-suppressive cytokines and soluble factors [12,42]. Here, we provide evidence that RV greatly reduced LPA-induced cell migration in three ovarian cancer cell lines, regardless of their mutated oncogenes and tumor suppressor genes, indicating that the mechanism though which RV suppresses cell motility is independent from the genetic background and the aggressiveness of the tumor.

The autophagic process is involved in the regulation of the epithelial-to-mesenchymal transition (EMT), a critical step in the process of cancer invasiveness. Depending on the context, autophagy plays a complex role by either inhibiting or promoting metastasis. On one side, autophagy hampers the early phases of metastasization by degrading EMT-associated markers [43] and reversing the EMT process [44], on the other hand it supports the survival of cancer cells that have already undergone EMT by preventing anoikis [45].

The transcriptomic analysis revealed that RV upregulates genes belonging to autophagy and apoptosis processes and strongly downmodulates the expression of transcripts involved in EMT, cell proliferation, glucose metabolism and several other oncogenic pathways associated with the invasive properties of ovarian cancer cells. RV effectively impaired the LPA-pro-migratory effect by raising the level of autophagy associated with re-expression of E-cadherin and repression of N-cadherin in the cells at the migration front.

Aberrant activation of the Hedgehog (Hh) pathway has been closely related to ovarian cancer cell proliferation, chemoresistance, stemness and EMT [46,47]. Consistently, we found that LPA and RV oppositely modulate the transcripts of the Hh pathway and of its downstream effectors. When compared to CP and GANT61, two well-known inhibitors of the Hh pathway, RV was demonstrated to be even more effective in counteracting LPA oncogenic activities, which included migration, cell proliferation, 3D spheroids growth and apoptotic response to oxaliplatin.

This benefit is attributable to the pleiotropic multi-target effects exerted by RV, as also indicated by the transcriptomic analysis. Mechanistically, LPA promoted malignancy through upregulation of the Hh and EMT pathways and concomitant inhibition of autophagy, and RV could counteract its effects by overcoming the inhibitory effect of BMI-1 and so rescuing autophagy.

BMI-1 is a polycomb ring finger transcription factor required for Hedgehog-promoted proliferation, metastasis and chemoresistance, and it correlates with poor prognoses in patients with ovarian cancer [35,36,48,49]. Interestingly, either genetically or pharmacologically targeting of BMI-1 has been shown to trigger autophagic cell death [50,51]. We demonstrated that *BMI-1* knockdown abrogates LPA-induced mesenchymal phenotype while upregulating autophagy in the cells at the migration front. Besides BMI-1, RV also downregulated the expression of GLI1, SNAIL-1 and TWIST1. These findings are in agreement with other reports proving the inhibitory activity of RV on the Hedgehog-GLI pathway in different cancers, supporting its adjuvant therapeutic potential to treat cancers with hyper-active Hedgehog signaling [52,53,54,55,56,57].

The stratification of ovarian cancer patients as poor and good responders to the platinum-based first-line therapy based on the oncogenic and tumor suppressor genetic background is crucial for implementation of personalized therapy [58]. Here we investigated whether the LPA-induced activation of the Hh pathway and of its downstream effectors (including GLI1, BMI-1, SNAIL-1, TWIST1) and the associated autophagy signature could have a translational relevance for therapeutic intervention. Interrogation of a TCGA dataset led to the conclusion that patients bearing a tumor with low expression of such Hh/EMT genes along with high expression of the *MAP1LC3B* gene (suggestive of active autophagy) have a better overall survival and are more responsive to platinum-based therapy. This finding is in agreement with the observation that low expression of IL-6 (indicative of stromal inflammation) along with high expression of LC3 (indicative of active autophagy) in cholangiocarcinomas associates with sensitivity to chemotherapy and better overall patient survival [59].

This prompted us to test *in vitro* the hypothesis that RV, like Hh inhibitors, could indeed have therapeutic potential by repressing the expression of BMI-1 and so rescuing autophagy and restoring sensitivity to OxPt cytotoxicity. The experiments conducted in the 3D spheroids model in fact proved that RV synergized with OxPt to induce BAX-mediated cell death in ovarian cancer cells in which BMI-1 was downregulated and LC3 was upregulated.

In conclusion, here we demonstrated a pivotal role of the Hh downstream effector BMI-1 in mediating the LPA pro-tumorigenic activities through inhibition of autophagy and the capability of RV to prevent Hh and BMI-1 activation and so rescuing autophagy and dampening the malignant features of ovarian cancer cells. We also show that ovarian cancer patients whose tumor express at low level Hh and EMT genes along with high expression of LC3 (indicative of active autophagy) better respond to platinum therapy and have longer survival. Finally, we prove that inhibition of BMI-1/Hh pathway along with stimulation of autophagy impairs the growth and sensitizes to platinum ovarian cancer cells. 

## 5. Conclusions

Taken together, this study has clinical relevance in terms of stratification of the ovarian cancer patients based on Hedgehog/EMT/autophagy gene signature for predicting responders and non-responders as well as of novel therapeutic strategies that include as adjuvants nutraceutical drugs that, like RV, act as autophagy enhancers and Hh inhibitors at the same time.

## Data Availability

Not applicable.

## Supplementary Materials

The following are available online at https://www.mdpi.com/article/10.3390/cells10113213/s1, Supplementary Table S1. Table reporting the genetic background and phenotypic characteristics of the three ovarian cancer cell lines used in the study. Note: * as published in [60]. N/A = not available information. Supplementary Figure S1. Low expression of Hedgehog/EMT markers predicts a good prognosis for ovarian cancer patients. Bioinformatic analysis performed on KM plotter ovarian cancer patients’ cohort (n = 1656). The overall survival curves were obtained based on *GLI1* (A), *BMI1* (B), *SNAI1* (C) and *TWIST1* (D) mRNA expression (high versus low). The log-rank test has been used to determine the statistical significance. The *p*-value ≤ 0.05 was considered as significant. Supplementary Figure S2. The expression of Hedgehog/EMT-related genes and of the autophagy marker *MAP1LC3B* are inversely correlated in ovarian cancer patients. The correlation analysis was performed on TCGA bioportal (ovarian serous cystadenocarcinoma dataset, TCGA Nature 2011) and the scatter plots obtained represent the relationship between *GLI1* (A), *BMI1* (B), *SNAI1* (C) and *TWIST1* (D) mRNA expression compared to *MAP1LC3B* levels. The number of patients sampled is reported (Observations). The Pearson’s correlation coefficient and *p*-value were used to determine statistical significance. The *p*-value ≤ 0.05 was considered as significant.

## Abbreviations

BMI-1, B-cell specific Moloney leukemia virus insertion site 1; CAFs, cancer-associated fibroblasts; CP, cyclopamine; DEGs, differentially expressed genes; EMT, epithelial-to-mesenchymal transition; GO, gene ontology; Hh, hedgehog pathway; LPA, lysophosphatidic acid; LPARs, LPA receptors; OxPt, oxaliplatin; RV, resveratrol; TCGA, The Cancer Genome Atlas; TME, tumor microenvironment.
