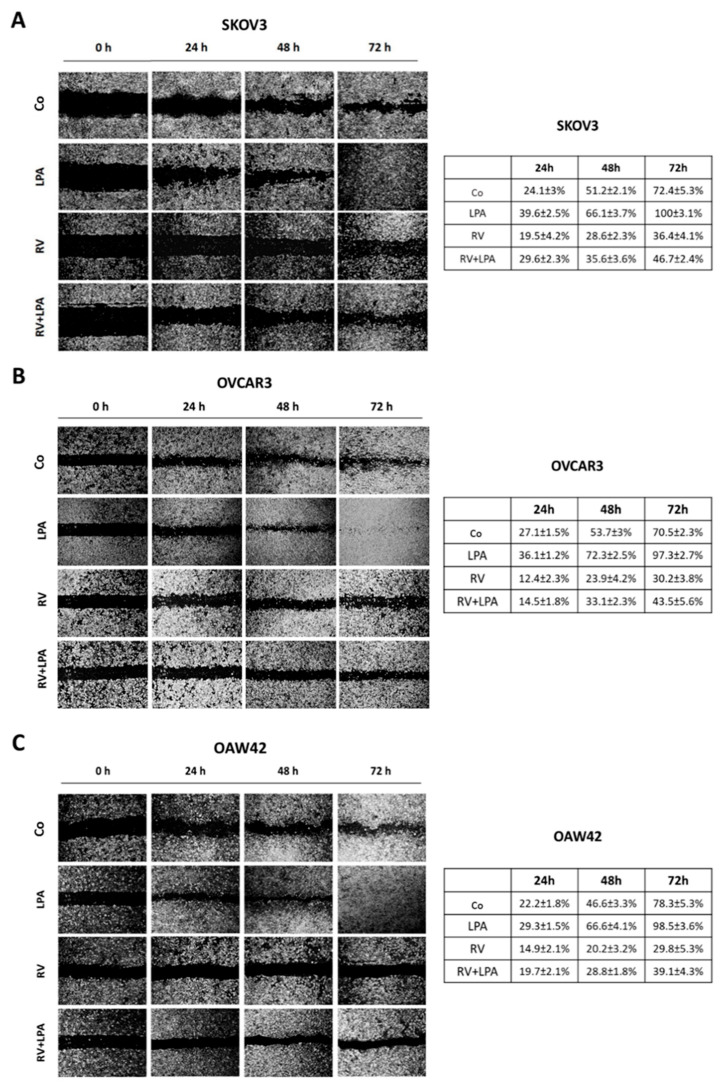# Correction: Ferraresi et al. Resveratrol Contrasts LPA-Induced Ovarian Cancer Cell Migration and Platinum Resistance by Rescuing Hedgehog-Mediated Autophagy. *Cells* 2021, *10*, 3213

**DOI:** 10.3390/cells14131020

**Published:** 2025-07-04

**Authors:** Alessandra Ferraresi, Andrea Esposito, Carlo Girone, Letizia Vallino, Amreen Salwa, Ian Ghezzi, Suyanee Thongchot, Chiara Vidoni, Danny N. Dhanasekaran, Ciro Isidoro

**Affiliations:** 1 Laboratory of Molecular Pathology, Department of Health Sciences, Università del Piemonte Orientale “A. Avogadro”, Via Solaroli 17, 28100 Novara, Italy; alessandra.ferraresi@med.uniupo.it (A.F.); andrea.esposito@uniupo.it (A.E.); carlo.girone@uniupo.it (C.G.); letizia.vallino@uniupo.it (L.V.); amreensalwa12@gmail.com (A.S.); 20020210@studenti.uniupo.it (I.G.); suyanee.thongchot@gmail.com (S.T.); chiara.vidoni@med.uniupo.it (C.V.); 2 Siriraj Center of Research Excellence for Cancer Immunotherapy (SiCORE-CIT), Research Department, Faculty of Medicine Siriraj Hospital, Mahidol University, Bangkok 10700, Thailand; 3 Stephenson Cancer Center, The University of Oklahoma Health Sciences Center, Oklahoma City, OK 73104, USA; danny-dhanasekaran@ouhsc.edu

## Error in Figure

In the original publication [1], there was a mistake in Figure 1 as published. The correction concerns panel B in Figure 1, where images of RV 24 and RV 48 h and LPA 48 h and LPA 72 h were duplicated due to an accidental technical error. The table reports the data (average ± S.D.) from three independent experiments performed by three different researchers (to guarantee reproducibility); the panel was incorrectly generated during the assembly of the figure. The corrected Figure 1 appears below. The authors state that the scientific conclusions are unaffected. This correction was approved by the Academic Editor. The original publication has also been updated.

**Figure:**