# Supplementary material for: Resveratrol Contrasts LPA-Induced Ovarian Cancer Cell Migration and Platinum Resistance by Rescuing Hedgehog-Mediated Autophagy
Source: Cells. 2021 Nov 17;10(11):3213. doi: 10.3390/cells10113213 (PMC8625920; doi:10.3390/cells10113213)
Supplement: Supplementary file 1 [file cells-10-03213-s001.zip › Supplementary files - cells-1456531 edited.pdf]

Supplementary Table 1. Table reporting the genetic background and phenotypic characteristics of the three ovarian cancer cell lines used in the study.

Cell line	Origin	Morphology	Grade	Platinum response	Genetic background						
					TP53	*PTEN*	*BECN1*	*BRCA1*	*PI3KCA*	*MYC*	*K-RAS*
SKOV3	Ovary adenocarcinoma, ascites	Spindle	2	Mild resistant	null	wt	wt	wt	wt	wt	wt
OVCAR3	Ovary epithelial carcinoma, ascites	Round	3	Resistant	mut	null	wt	wt	mut	wt	wt
OAW42	Ovary mucinous cystoadenocarcinoma, ascites	Round	N/A	Sensitive	wt	wt (methylated*)	wt (low expression)	wt	mut	wt	wt

Note:

* as published in [60]

N/A = not available information

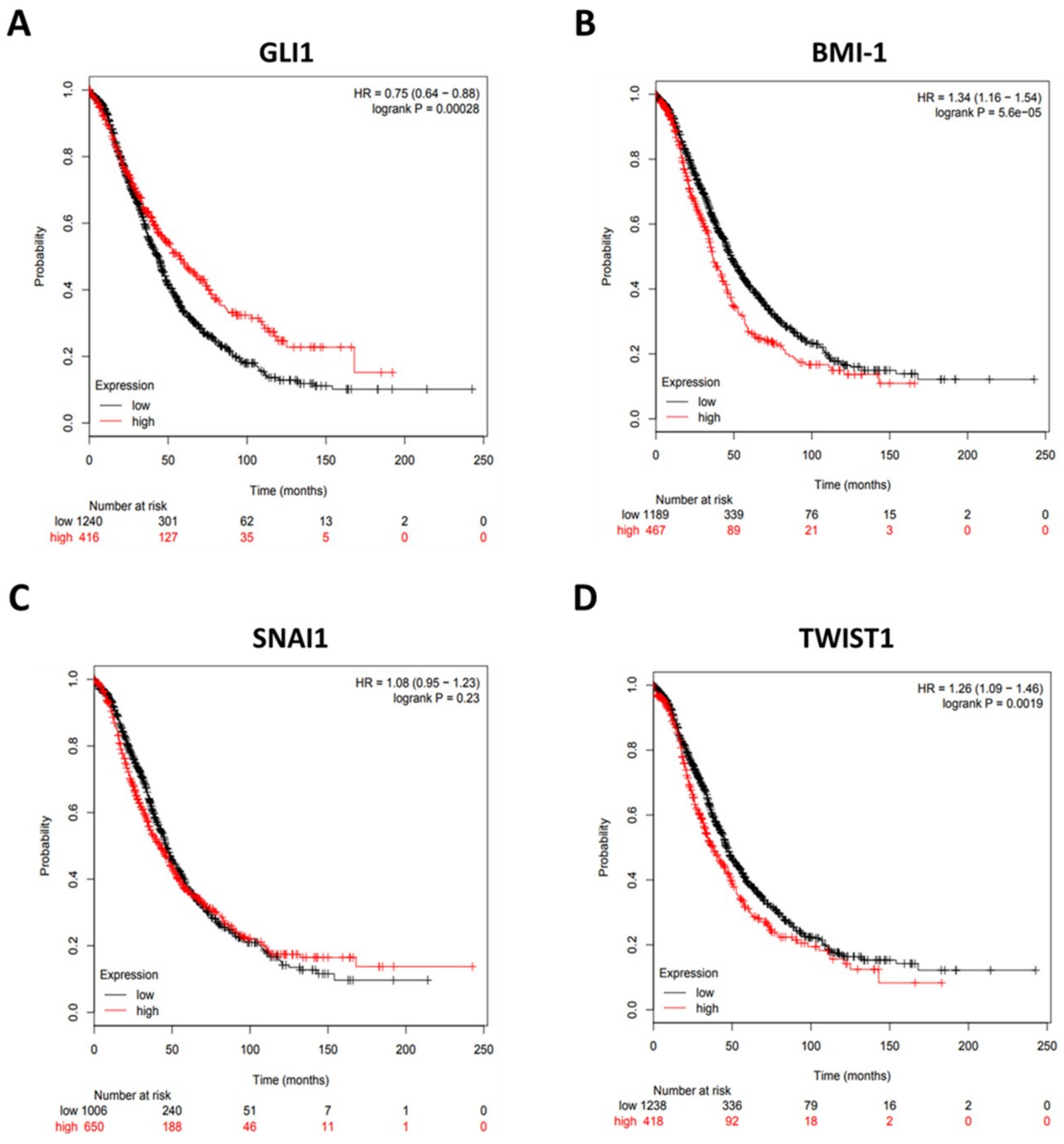

Supplementary Figure S1. Low expression of Hedgehog/EMT markers predicts a good prognosis for ovarian cancer patients.

Bioinformatic analysis performed on KM plotter ovarian cancer patients' cohort (n=1656). The overall survival curves were obtained based on *GLI1* (**A**), *BMI1* (**B**), *SNAI1* (**C**) and *TWIST1* (**D**) mRNA expression (high *versus* low). The log-rank test has been used to determine the statistical significance. The p-value ≤ 0.05 was considered as significant.

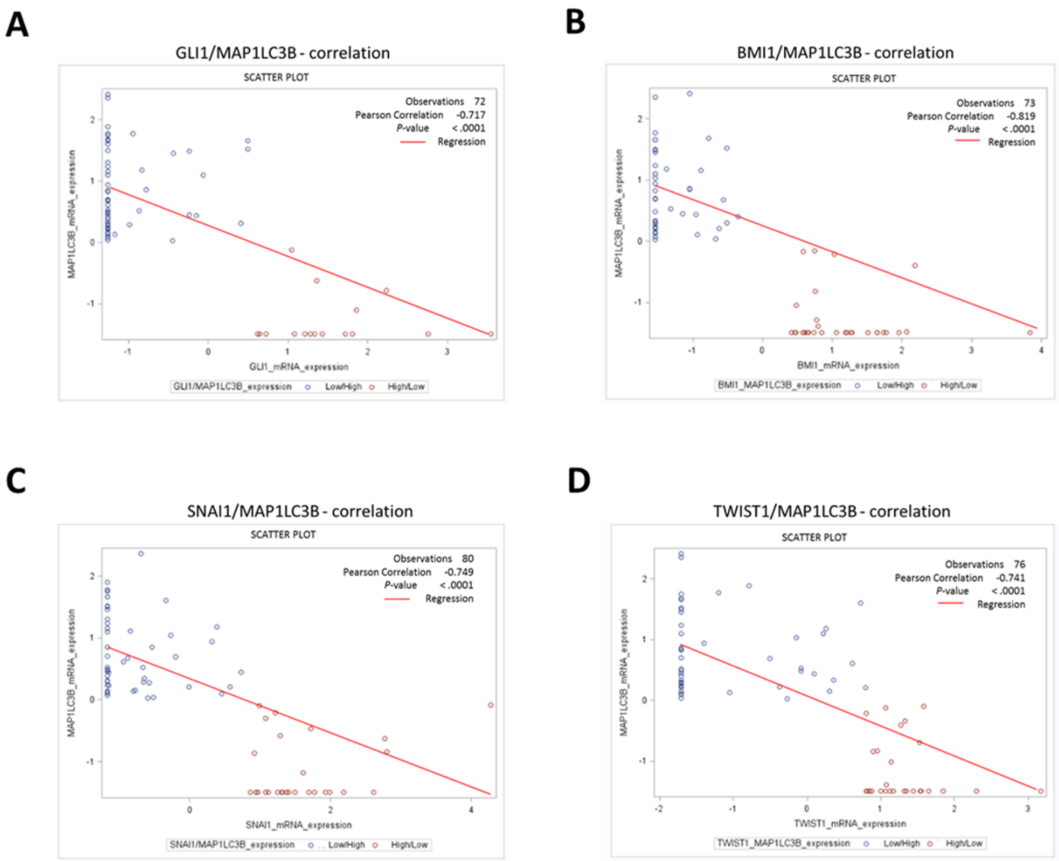

Supplementary Figure S2. The expression of Hedgehog/EMT-related genes and of the autophagy marker MAP1LC3B are inversely correlated in ovarian cancer patients.

The correlation analysis was performed on TCGA bioportal (ovarian serous cystadenocarcinoma dataset, TCGA Nature 2011) and the scatter plots obtained represent the relationship between *GLI1* (**A**), *BMI1* (**B**), *SNAI1* (**C**) and *TWIST1* (**D**) mRNA expression compared to *MAP1LC3B* levels. The number of patients sample is reported (Observations). The Pearson's correlation coefficient and p-value were used to determine statistical significance. The p-value ≤ 0.05 was considered as significant.
